# Supplementary material for: NSUN2 introduces 5-methylcytosines in mammalian mitochondrial tRNAs
Source: Nucleic Acids Res. 2019 Jul 5;47(16):8720–33. doi: 10.1093/nar/gkz559 (PMC6822013; doi:10.1093/nar/gkz559)
Supplement: gkz559_Supplemental_File [file gkz559_supplemental_file.pdf]

SUPPLEMENTARY DATA

NSUN2 introduces 5-methylcytosines in mammalian mitochondrial tRNAs

Lindsey Van Haute *et al*.

CONTENTS

Supplementary Figure S1 | Generation and characterisation of NSUN2 CRISPR/Cas9 KO cells – page 2

Supplementary Figure S2 | Detailed MS/MS spectra of NSUN2 DBP-peptides – page 3

Supplementary Figure S3 | Transient expression of NSUN2 in human NSUN2 KO cells – page 4

Supplementary Table S1 | List and sequences of gRNA used to generate human NSUN2 KO line – page 5

Supplementary Table S2 | List of oligonucleotides used in this study - pages 6-8

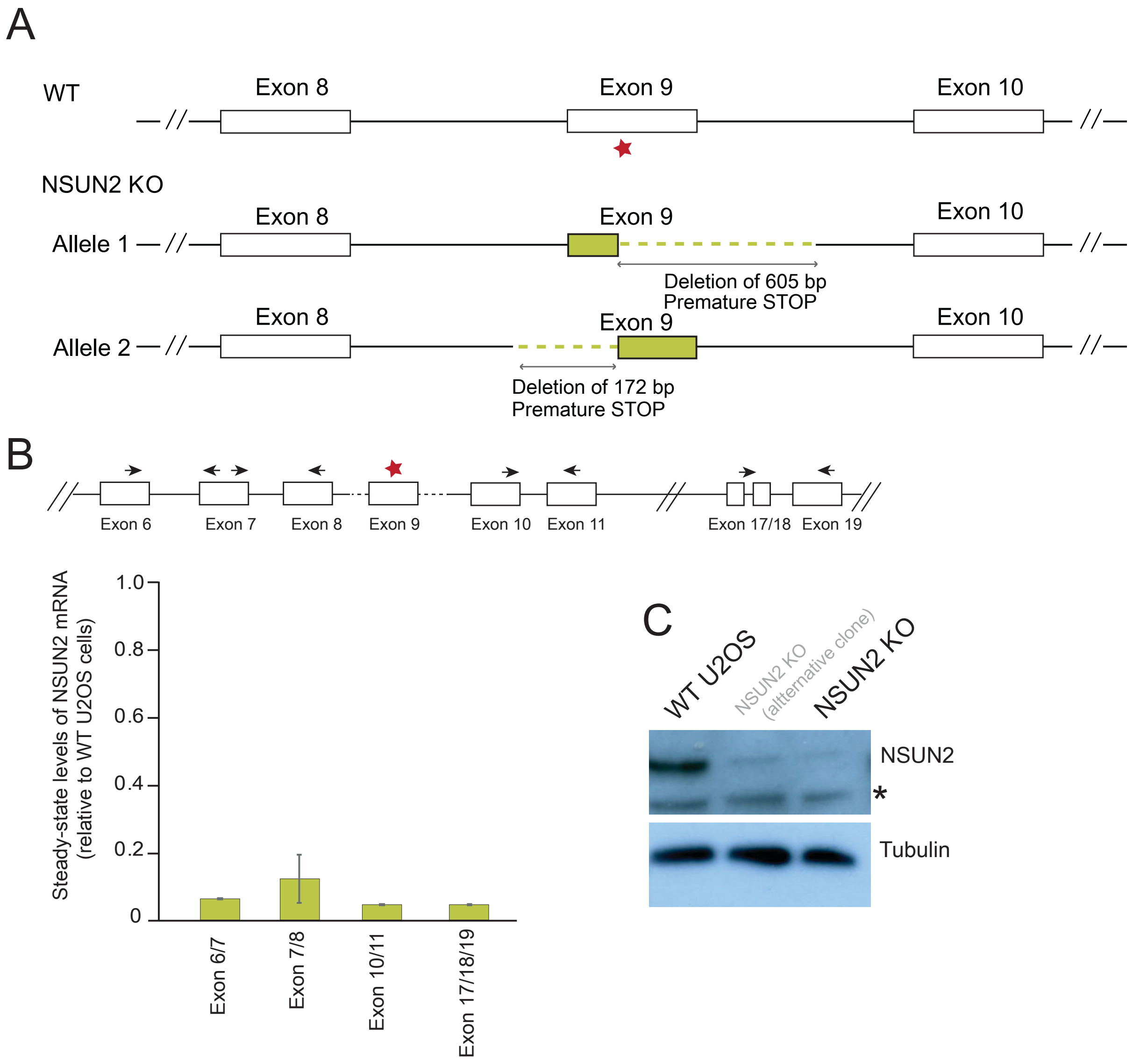

Supplementary Figure S1 | Generation and characterisation of NSUN2 CRISPR/Cas9 KO cells

(**A**) Schematic representation of a fragment of the NSUN2 gene structure spanning exons 8 to 10. Red star indicates the site in exon 9, which was targeted by the CRISPR/Cas9 gRNAs (**Supplementary Table S1**). Genomic changes in the U2OS NSUN2 KO cell line as detected by PCR and Sanger sequencing are indicated in green and described. (**B**) *Top*: the NSUN2 gene structure spanning exons 6 to 19 with indicated RT-qPCR primer binding sites. *Bottom*: RT-qPCR analysis of NSUN2 mRNA expression levels in the NSUN2 KO cells for 4 pairs of primers. The expression was normalised to GAPDH. n=2. (**C**) Western blot analysis of the steady-state levels of NSUN2 in U2OS WT and NSUN3 KO cell line. Note: alternative CRISPR/Cas9 KO line is shown, which was not used in the present study.

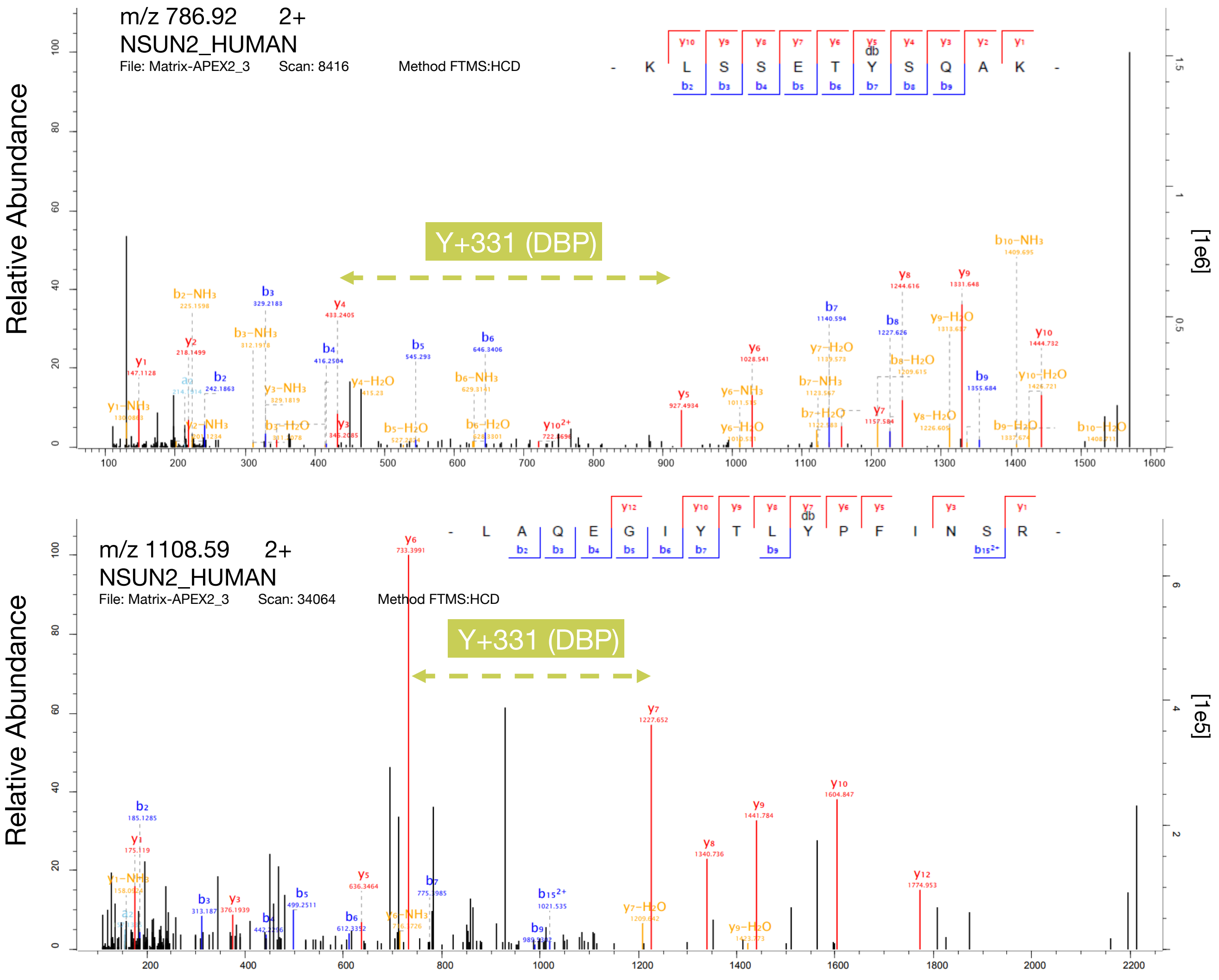

Supplementary Figure S2 | Detailed MS/MS spectra of NSUN2 DBP-peptides. Spectra for KLSSETYSQAK and LAQEGIYTLYPFINSR (DBP-modified Y underlined) labelled by mitochondrial matrix-targeted APEX2.

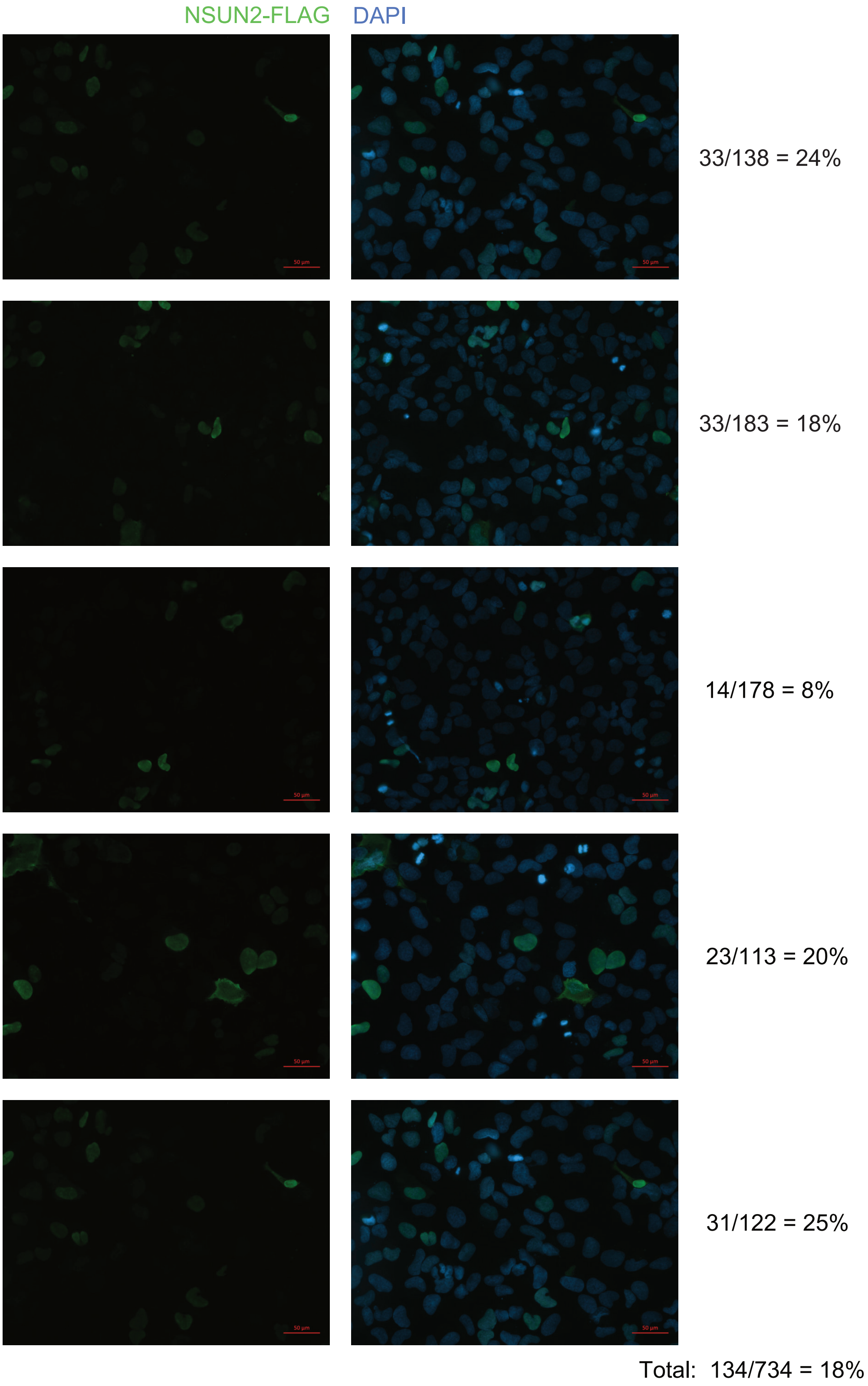

Supplementary Figure S3 | Transient expression of NSUN2 in human NSUN2 KO cells

Representative images of immunofluorescence labelling after transient expression of a NSUN2.FLAG.STREP2 construct in human NSUN2 KO cells. Cells were stained for FLAG (green) and DAPI (blue). Scale bar: 50μM. Average percentage of transfected cells was 18 %.

Supplementary Table S1 | List and sequences of gRNA used to generate human NSUN2 KO line

**gRNA name**	**gRNA sequence**
gRNA_C321A_1_fwd	pACCGCAATCCGCAGCTGTAAGCTA
gRNA_C321A_1_rev	pAAACTAGCTTACAGCTGCGGATTG
gRNA_C321A_2_fwd	pACCGGTGTTCACTAAACCCTATTG
gRNA_C321A_2_rev	pAAACCAATAGGGTTTAGTGAACAC

Supplementary Table S2 | List of oligonucleotides used in this study

Purpose		5' to 3' sequence
**Primers used for cloning into pcDNA5-FST2**		
NSUN2 start	forward	GACGGTACCATGGGGCGGCGGTCGCGGGG
NSUN2 stop	reverse	GTCCTCGAGCCGGGGTGGATGGACCCCC
**qPCR primers for DNA analysis**		
mt-CO1	forward	TGCTAGCCGCAGGCATTACT
	reverse	CGGGATCAAAGAAAGTTGTGTTT
RNaseP	forward	GCCTACACTGGAGTCCGTGCTACT
	reverse	CTGACCACACACGAGCTGGTAGAA
**qPCR primers for RT-qPCR**		
NSUN2 ex17-18	forward	AAGCAAAGGACCTGGCAAAG
NSUN2 ex19	reverse	CAGCCCCATCATCCTGAGAT
NSUN2 ex10	forward	AGTGGATGCCTGGAATCACA
NSUN2 ex11	reverse	GGGAACATGGTAGGTCGGAT
NSUN2 ex6	forward	CTGGCTCAAAGACCACACAG
NSUN2 ex7	reverse	GTTGACCACCATGATGCAGG
NSUN2 ex7	forward	CCTGCATCATGGTGGTCAAC
NSUN2 ex8	reverse	TCTCATAGTGCCGTCTCCAC

Supplementary Table S2 - continued

Targeted RNA BS-seq primers human		
hMT-TS2 RT primer		TAAAAAAACCATATTATTAAACA
hMT-TS2 1stage PCR primer with overhang	forward	TCGTCGGCAGCGTCAGATGTGTATAAGAGACAGGTTTATAAGAATTGTTAATTTATG
hMT-TS2 1stage PCR primer with overhang	reverse	GTCTCGTGGGCTCGGAGATGTGTATAAGAGACAGTAAAAAAACCATATTATTAAACA
hMT-TM RT primer		TTAGTTAAATAAGTTATTGGGT
hMT-TM 1stage PCR primer with overhang	forward	TCGTCGGCAGCGTCAGATGTGTATAAGAGACAGTTAGTTAAATAAGTTATTGGGT
hMT-TM 1stage PCR primer with overhang	reverse	GTCTCGTGGGCTCGGAGATGTGTATAAGAGACAGAACCAACATTTTCAAAATA
hMT-TH RT primer		TAAAAATCATAAACCTC
hMT-TH 1stage PCR primer with overhang	forward	TCGTCGGCAGCGTCAGATGTGTATAAGAGACAGTTAAAATATTAGATTGTGAATTTG
hMT-TH 1stage PCR primer with overhang	reverse	GTCTCGTGGGCTCGGAGATGTGTATAAGAGACAGAATAAAAAATCATAAACCTC
hMT-TL1 RT primer		TATTAAAAAAAAAAATTAAACCTC
hMT-TL1 1stage PCR primer with overhang	forward	TCGTCGGCAGCGTCAGATGTGTATAAGAGACAGGGTAATTGTATAAAATTTAAAAT
hMT-TL1 1stage PCR primer with overhang	reverse	GTCTCGTGGGCTCGGAGATGTGTATAAGAGACAGTATTAAAAAAAAAAATTAAACCTC
hMT-TE RT primer		TATTCTCACACAAACTACAACCA
hMT-TE 1stage PCR primer with overhang	forward	TCGTCGGCAGCGTCAGATGTGTATAAGAGACAGAATATAATGATGGTTTTTTATA
hMT-TE 1stage PCR primer with overhang	reverse	GTCTCGTGGGCTCGGAGATGTGTATAAGAGACAGTATTCTCACACAAACTACAACCA
hMT-TF RT primer		TATTTATAAAATAATATAAACC
hMT-TF 1stage PCR primer with overhang	forward	TCGTCGGCAGCGTCAGATGTGTATAAGAGACAGTAAAGTAATATATTGAAAATGTTT
hMT-TF 1stage PCR primer with overhang	reverse	GTCTCGTGGGCTCGGAGATGTGTATAAGAGACAGTATTTATAAAATAATATAAACC
hMT-TY RT primer		ATAATAAAAAAAAACCTAACCCC
hMT-TY 1stage PCR primer with overhang	forward	TCGTCGGCAGCGTCAGATGTGTATAAGAGACAGGTTGAGTGAAGTATTGGATTGTAA
hMT-TY 1stage PCR primer with overhang	reverse	GTCTCGTGGGCTCGGAGATGTGTATAAGAGACAGATAATAAAAAAAAACCTAACCCC

Supplementary Table S2 - continued

**Targeted RNA BS-seq primers mouse**		
mMT-TH RT primer		AATAAATAAAAAAATTTATTTCC
mMT-TH 1stage PCR primer with overhang	forward	TCGTCGGCAGCGTCAGATGTGTATAAGAGACAGAAAATATTAGATTGTGAATTTG
mMT-TH 1stage PCR primer with overhang	reverse	GTCTCGTGGGCTCGGAGATGTGTATAAGAGACAGAATAAATAAAAAAATTTATTTCC
mMT-TL1 RT primer		TATTAAAAAGAAAATTTAAACCTC
mMT-TL1 1stage PCR primer with overhang	forward	TCGTCGGCAGCGTCAGATGTGTATAAGAGACAGTGTGTAAGATTTAAAATTTTGT
mMT-TL1 1stage PCR primer with overhang	reverse	GTCTCGTGGGCTCGGAGATGTGTATAAGAGACAGTATTAAAAAGAAAATTTAAACCTC
mMT-TL2 RT primer		TACTTTTATTTAAATTTACACCA
mMT-TL2 1 stage PCR primer with overhang	forward	TCGTCGGCAGCGTCAGATGTGTATAAGAGACAGATAATAGTAATTTATTGGTTTTAGGA
mMT-TL2 1stage PCR primer with overhang	reverse	GTCTCGTGGGCTCGGAGATGTGTATAAGAGACAGTACTTTTATTTAAATTTACACCA
mMT-TY RT primer		TAATAAAAAAAAAATTTAAACCTC
mMT-TY 1 stage PCR primer with overhang	forward	TCGTCGGCAGCGTCAGATGTGTATAAGAGACAGGAGTAAGTATTAGATTGTAAAT
mMT-TY 1 stage PCR primer with overhang	reverse	GTCTCGTGGGCTCGGAGATGTGTATAAGAGACAGTAATAAAAAAAAAATTTAAACCTC
mMT-TN RT primer		CTAAATTAACAAAAATTAAACCTA
mMT-TN 1 stage PCR primer with overhang	forward	TCGTCGGCAGCGTCAGATGTGTATAAGAGACAGTAATAGGGTATTTAGTTGTTAA
mMT-TN 1 stage PCR primer with overhang	reverse	GTCTCGTGGGCTCGGAGATGTGTATAAGAGACAGCTAAATTAACAAAAATTAAACCTA
mMT-TS2 RT primer		TAAAAAAACCATATTTTTAAACA
mMT-TS2 1stage PCR primer with overhang	forward	TCGTCGGCAGCGTCAGATGTGTATAAGAGACAGTTGTAAGAATTGTTAATTTATG
mMT-TS2 1stage PCR primer with overhang	reverse	GTCTCGTGGGCTCGGAGATGTGTATAAGAGACAGTAAAAAAACCATATTTTTAAACA
mMT-TE RT primer		TATTTCTACACAACATTCAACTA
mMT-TE 1stage PCR primer with overhang	forward	TCGTCGGCAGCGTCAGATGTGTATAAGAGACAGTGATGATTTTTTATGTTATTGG
mMT-TE 1stage PCR primer with overhang	reverse	GTCTCGTGGGCTCGGAGATGTGTATAAGAGACAGTATTTCTACACAACATTCAACTA
